# Optimal Experimental Design for Parameter Estimation of a Cell Signaling Model

**DOI:** 10.1371/journal.pcbi.1000558

**Published:** 2009-11-06

**Authors:** Samuel Bandara, Johannes P. Schlöder, Roland Eils, Hans Georg Bock, Tobias Meyer

**Affiliations:** 1 Department of Chemical and Systems Biology, Stanford University, Stanford, California, United States of America; 2 Interdisciplinary Center for Scientific Computing, University of Heidelberg, Heidelberg, Germany; 3 Theoretical Bioinformatics, German Cancer Research Center, Heidelberg, Germany; 4 Institute of Pharmacy and Molecular Biotechnology/BIOQUANT, University of Heidelberg, Heidelberg, Germany; ETH Zurich, Switzerland

## Abstract

Differential equation models that describe the dynamic changes of biochemical signaling states are important tools to understand cellular behavior. An essential task in building such representations is to infer the affinities, rate constants, and other parameters of a model from actual measurement data. However, intuitive measurement protocols often fail to generate data that restrict the range of possible parameter values. Here we utilized a numerical method to iteratively design optimal live-cell fluorescence microscopy experiments in order to reveal pharmacological and kinetic parameters of a phosphatidylinositol 3,4,5-trisphosphate (PIP_3_) second messenger signaling process that is deregulated in many tumors. The experimental approach included the activation of endogenous phosphoinositide 3-kinase (PI3K) by chemically induced recruitment of a regulatory peptide, reversible inhibition of PI3K using a kinase inhibitor, and monitoring of the PI3K-mediated production of PIP_3_ lipids using the pleckstrin homology (PH) domain of Akt. We found that an intuitively planned and established experimental protocol did not yield data from which relevant parameters could be inferred. Starting from a set of poorly defined model parameters derived from the intuitively planned experiment, we calculated concentration-time profiles for both the inducing and the inhibitory compound that would minimize the predicted uncertainty of parameter estimates. Two cycles of optimization and experimentation were sufficient to narrowly confine the model parameters, with the mean variance of estimates dropping more than sixty-fold. Thus, optimal experimental design proved to be a powerful strategy to minimize the number of experiments needed to infer biological parameters from a cell signaling assay.

## Author Summary

Differential equation models of signaling processes are useful to gain a molecular and quantitative understanding of cellular information flow. Although these models are typically based on simple kinetic rules, they can often qualitatively describe the behavior of biological systems. However, in the quest to transform biomedical research into an engineering discipline, biologists face the challenge of estimating important parameters of such models from laboratory data. Measurement noise as well as the robust architecture of biological circuits are causes for large uncertainty of parameter estimates. This makes it difficult to plan informative experiments. Here, we used a computational method to predict and minimize the uncertainty of parameter estimates we would obtain from prospective experiments given a cancer-relevant signaling model. This was achieved by optimizing the concentrations and time points for adding drugs in a live-cell microscopy experiment. Our experimental results demonstrated that the advice given by this algorithm resulted in many-fold more informative data than we would obtain by repeating an intuitively planned experiment. Our study shows that significant experimental effort and time can be saved by adopting an optimal experimental design strategy for inferring relevant parameters from biomedical experiments.

## Introduction

Biological cells continuously process stimuli that they receive from their inside and outside, using interconnected signaling pathways. Since strength, timing, and combination of inputs typically determine the output behavior in a non-linear manner, dynamic models of such signaling networks in the form of differential equations are becoming widely used to complement experimental studies [1],[2]. A major challenge to systems biology is therefore, to infer the rate constants, binding affinities and other parameters of such models from actual measurement data [3],[4]. Parameter estimation is a widely applied method for model calibration in mechanical and chemical engineering, and — if transferred to the field of cell signaling — may facilitate the development of predictive models of disease and therapy [5]. However, because measurements are always afflicted with error, parameter estimates which minimize the discrepancy between model and data often come along with high uncertainty. Gutenkunst *et al.* [6] studied parameter sensitivities in 17 published models in the field of systems biology, and concluded that parameter estimation in biology is difficult not only because of often large measurement error, but also because model behavior is often insensitive to combined changes of parameter values. The resulting working models thus remain limited in mechanistic insight and in their capability to predict system dynamics in unforeseen conditions.

For a given set of differential equations that describes a dynamic process, not every experimental protocol is equally suited to produce data from which parameters can be inferred. However, it is often possible to control a dynamic process in such a way that predicted measurements are very sensitive to the parameter values that underlie the dynamic system. In that case, the data would be better suited for parameter estimation because it restricts the range of parameter values that could explain the data. To efficiently determine experimental protocols that sensitize model predictions to parameter values, optimal control problems need to be solved. Körkel *et al.* [7],[8] developed and implemented an efficient scheme for solving such optimal control problems in chemical engineering, given deterministic models in the form of ordinary differential equations (ODE) or differential algebraic equations (DAE). These solutions propose how to best feed a reactor, control its temperature, and when to take measurements in order to estimate parameters with highest accuracy, while obeying constraints imposed by feasibility or cost. Although the computed experimental protocols and sampling schemes are unavoidably based only on approximate models and parameter values, experience shows that data evaluated from optimized experiments are typically more informative than data from intuitively planned experiments[8]. By designing experiments sequentially, the iterative process of performing experiments can be paralleled by an increasing predictive power to plan the next experiment.

Several computational studies on models of the MAP kinase cascade, apoptosis, STAT5 activation, and the 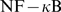 pathway [9]–[14] suggested that this strategy could also be valuable for the experiment-driven modeling of signal transduction networks. These studies showed that given a specific model of a biological process, several numerical methods can be used to identify stimulus and sampling patterns that would reveal parameter values with higher accuracy. The practical value of such an approach could be immense, because experiments are sensitized to reveal otherwise hidden biological parameters. However, despite a wealth of diverse numerical approaches [8],[9],[11],[13],[15] (reviewed in [16]), those methods have not been adopted by experimentalists. The theoretical nature of previous work left it unclear, whether optimized experiments would drive a true biological system within the predictive range of a model [15], how experimental uncertainty and imperfection affect the attainable accuracy of estimates, and how design performance is affected by the possibly large discrepancy between true biology and a differential equations model. Moreover, the increased complexity of proposed stimulus designs has raised the question whether numerically optimized experiments are even feasible enough to return net savings of experimental effort in a real-world cell biology lab.

**Figure 1:**
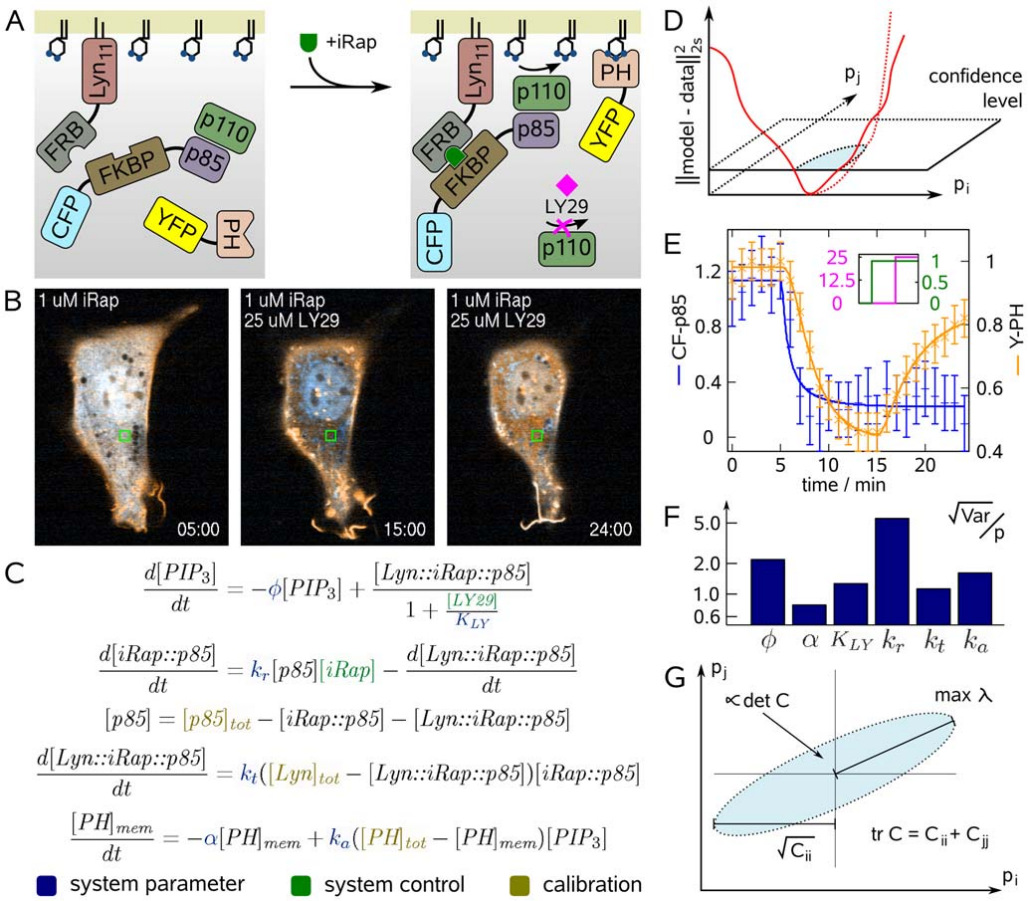
Parameter estimation of PI3K signaling reveals identifiability problems for important kinetic properties. (A) iRap-induced dimerization of FKBP with FRB recruits CF-p85 to the plasma membrane and activates the catalytic subunit p110 of PI3K to produce 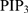 from 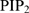. PH domains of many downstream signaling proteins bind to 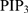 enriched membranes with differential affinities. LY29 is a widely used inhibitor of PI3K. (B) Confocal fluorescence images of an NIH 3T3 cell (CF-p85 shown in cyan, Y-PH in yellow). Initially, CF-p85 and Y-PH reside in the cytosol (left). Addition of iRap recruits both constructs to the plasma membrane as can be judged from cytosolic depletion (middle). Y-PH but not CF-p85 dissociates from the plasma membrane upon addition of LY29. Experiment time is shown in minutes. (C) Differential equations model of the dynamic system illustrated in (A). System parameters are shown in blue, variables that allow control of the system are shown in green, and calibration parameters which were different from cell to cell are shown in gold. (D) Parameter values can be inferred by minimizing the discrepancy between model and experimental data. The schematic illustrates this concept for two parameters, 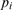 and 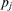. The range of possible parameter values is where this discrepancy falls below the confidence level (blue shaded ellipsoid). If this range is large, parameters can be inferred only with high uncertainty. (E) Fluorescence was quantified in the green square depicted in (B), and the model in (C) was fit to that data. Error bars show data, continuous lines show model output. Blue error bars and lines show cytosolic fluorescence of CF-p85, orange error bars and lines cytosolic fluorescence of Y-PH. (F) The relative uncertainty of parameter estimates was very high. This is a common problem in systems biology models. (G) Schematic projection of the confidence ellipsoid from (D). Good experiments minimize the size of this uncertainty region (shaded blue). Criteria that have been proposed for describing this size are the longest half-axis, the average extent, or the volume of the confidence ellipsoid, among others. These criteria correspond to properties of the parameter covariance matrix 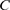.

Here, we addressed these questions explicitly by employing a numerical approach to enrich an existing single-cell assay for pharmacologically, biochemically, and clinically relevant parameters by an optimized protocol. Our study focused on the accumulation of the phosphatidylinositol 3,4,5-trisphosphate (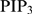) second messenger lipid and the subsequent recruitment of downstream signaling elements through pleckstrin homology (PH) domains. 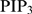 is produced by the catalytic subunit p110 of phosphoinositide 3-kinase (PI3K) in response to chemokine or other receptor stimuli in the plasma membrane of eukaryotic cells. Slow diffusion and rapid degradation of 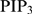 result in gradients that are steep enough to mediate cell polarity as in migration and differentiation, but elevated synthesis of 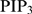 is associated with cancer. Signaling elements downstream of PI3K, like the oncogenic Akt protein, engage by binding to 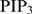-enriched membranes through pleckstrin homology (PH) domains. Previously, Suh *et al.* [17] described a chemical method for activating endogenous p110 at the plasma membrane. This technique makes use of the rapamycin-dependent heterodimerization of the FK506-binding protein (FKBP) with the mammalian target of rapamycin (mTOR). A genetic fusion of cyan fluorescent protein (CFP), FKBP12, and a peptide from the regulatory subunit p85 of PI3K was constructed (CF-p85) that resides in the cytosol and does not intrinsically stimulate p110 activity. However, upon addition of the rapamycin derivative iRap to cells co-transfected with a construct made from the N-terminal plasma membrane-targeting sequence of Lyn and the FKBP-rapamycin binding domain of mTOR (Lyn-FRB), CF-p85 translocates to the plasma membrane and induces the production of 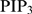 (Figure 1A). Elevated concentrations of 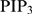 can be monitored through translocation events of a construct in which yellow fluorescent protein is fused to the PH-domain of Akt (Y-PH).

Our experimental setup observed translocation dynamics of CF-p85 and Y-PH in NIH 3T3 fibroblasts by live-cell confocal fluorescence microscopy in order to infer the kinetic parameters of the underlying dynamic system. If such parameters were inferable with high statistical confidence, it would be possible, for example, to compare the inhibitory effects of different cancer drug candidates on PI3K directly and *in situ*. To shed light on nonlinear signal transmission, it would be desirable also to reveal differential affinities of the more than 20 PH domains that were shown to be 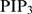 responsive [18]. Moreover, the degradation of 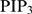 is compromised in many tumors, whereas in others, sustained growth factor inputs could be responsible for elevated downstream signaling. We asked if the established single-cell assay would yield dynamic data from which such relevant properties could be inferred by fitting a differential equations model. However, we found that our intuitively planned and often used experimental protocol was not informative about these parameters. Even for a simplified model, the parameter estimates were largely undefined and covaried strongly. Since the traditional experimental protocol involved two drugs, iRap and the PI3K inhibitor LY294002 (LY29), we utilized a numerical optimization method [7],[8] to design better concentration-time profiles for these drugs in order to reveal model parameters with higher accuracy. We performed these numerically optimized experiments sequentially, and found that the uncertainty of parameter estimates could be reduced dramatically and covariance be largely eliminated. Given the potential implications on systems biology methodology, the paper derives meaningful constraints of the optimization problem as it pertains to live-cell experimentation, and investigates the implications of experimental uncertainty and imperfection.

## Results

NIH 3T3 fibroblasts were transfected with three constructs, (i) a plasma membrane-targeted FRB domain (Lyn-FRB), (ii) an inducible PI3K-activating peptide derived from p85 and conjugated to an FKBP12 domain and CFP (CF-p85), and (iii) a YFP-conjugated PH domain from Akt as a biosensor for 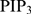 (Y-PH). Because growth factors present in serum already activate PI3K, the cells were serum-starved before undergoing live imaging by confocal fluorescence microscopy. Throughout the first 5 min of imaging, the cells were not perturbed, and both CF-p85 and Y-PH resided in the cytosol (Figure 1B and Video S1). After addition of 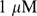 iRap, CF-p85 rapidly translocated to the plasma membrane, closely followed by Y-PH. The translocation of these constructs indicates that iRap-mediated heterodimerization of the FRB and FKBP12 domains recruits the p85-derived peptide to the plasma membrane where it triggers PI3K to produce 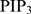. The accumulation of 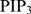 in turn traps Y-PH at the plasma membrane, as indicated by a decline in cytosolic fluorescence. Ten minutes after the addition of iRap, the production of 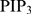 was effectively halted by the addition of 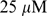 LY29. Y-PH immediately started to dissociate from the plasma membrane and a marked increase in cytosolic fluorescence of Y-PH was observed, suggesting rapid turnover of 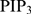.

### Inferring kinetic and pharmacological properties from single-cell data

In order to infer biological parameters from the observed translocation dynamics, we set up a differential equations model (Figure 1C, Text S1) that reflects this system. It is a system of ordinary differential equations (ODE) of the form
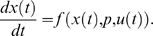
(1)It comprises differential states 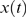 for the amounts of 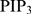 (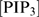), of CF-p85 bound to iRap (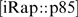), of CF-p85 in a ternary complex with Lyn-FRB via iRap (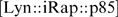) and of Y-PH bound to the plasma membrane (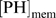). All other molecular species were derived from total amounts via mass balance. The parameters 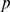 of this model are the rate constant 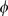 for the degradation of 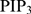, the inhibitor constant 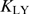 for the effect of LY29 on p110, the rate constants 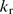 and 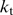 for the stepwise formation of the ternary complex, 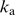 for the attachment of Y-PH to 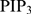-enriched membranes, and 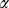 for its dissociation (Figure 1C, blue). This simplified model assumes that the formation of the complex of CF-p85, iRap, and Lyn-FRB begins with the binding of iRap to CF-p85 [19], that both this reaction and the subsequent binding of Lyn-FRB are irreversible, and that Y-PH does not shield 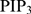 from degradation. Because the amounts of the exogenously expressed CF-p85, Lyn-FRB, and Y-PH vary from experiment to experiment 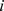, the total amount of each of those species 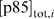, 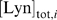, and 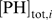 were additional model parameters that solely served the purpose of calibration (Figure 1C, gold). The time-dependent concentrations of iRap and LY29 were represented as piecewise constant functions 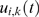 stepping from 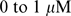 at 5 min for iRap, and from 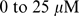 for LY29 10 min later:
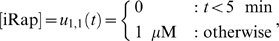
(2)

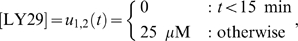
(3)where 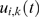 denotes the concentration of the respective drug 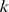 in experiment 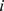 at time 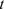 (Figure 1C, green).

The cytosolic fluorescence intensities of CF-p85 and Y-PH were measured for a single cell from 25 frames captured every minute. In order to relate the measured values 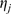 from frame 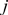 to a cytosolic concentration in the model at time 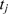, the observation functions
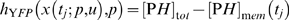
(4)

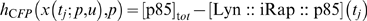
(5)were defined. Using these observation functions, the model can be fitted to the two fluorescence trajectories by finding a parameter set 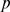 that minimizes a weighted least squares functional:
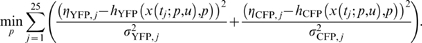
(6)The measurement errors 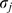 were estimated from individual frames (see Methods). For collective fits from 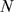 independent experiments 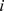, the least squares functional is a sum over 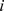 of the above expression (6).

Figure 1D shows a schematic of the parameter estimation concept. For the experimental treatment described by 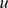, the model predicts observations 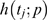 that should coincide — within the range of measurement error — with data 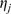. If the predicted observations depend on the model parameters 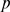, it is possible to infer the parameter set that minimizes the discrepancy between model and data. If this discrepancy falls below the confidence level which is defined by the measurement errors 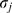, the model is considered a “fit”. To infer parameter values from microscopy experiments, i.e. to minimize expression (6), a multiple shooting Gauss-Newton type algorithm was employed (see Methods). The resulting parameter set yielded good agreement between model and data (Figure 1E).

However, the behavior of biological systems is often robust to changes in rate constants, binding affinities, and concentrations. Thus, large regions in parameter space often correspond to similarly good model predictions, and therefore, it is often not possible to infer parameter values from experimental data with satisfying precision [6]. Figure 1D depicts this uncertainty region as the area where the least squares functional falls below the confidence level hyperplane (blue shaded region). In other words, the uncertainty of parameter estimates is inversely related to the sensitivity of testable model predictions 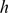 to changes in parameter values 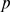:
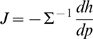
(7)where 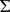 is the matrix of diagonalized 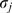 and 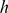 are the vectorized predictions 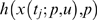 of the ODE model. From these sensitivities, the parameter covariance matrix 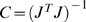 can be obtained. Note that the covariance matrix 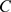 depends on the experimental protocol 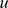. 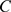 describes the uncertainty region in parameter space in proximity of the solution 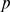 of the estimation problem. In particular, the 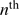 diagonal element of this matrix approximates the variance 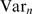 of a specific parameter estimate 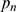. For the initial fit shown in Figure 1E, the relative uncertainties 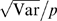 indicate that the intuitive experimental protocol is not very suitable to define the parameters of interest (Figure 1F, Table 1).

**Table 1:** Parameter estimates and their uncertainties.

Parameter	after traditional		after 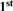 optimized		after 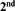 optimized	
	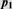	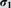	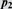	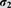	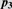	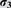
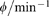	1.88	4.10	5.23	11.91	1.80	1.95
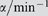	0.23	0.18	0.16	0.03	0.11	0.03
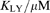	3.67	4.66	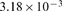	0.46	0.16	0.34
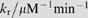	6.09	33.45	1.03	0.85	1.56	1.33
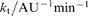	1.47	1.65	3.82	5.44	0.03	3.41
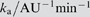	0.59	0.96	1.24	2.71	0.58	0.52
**residual**	8.34		10.30		11.15	
**D-criterion** 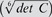	0.87		0.31		0.18	
**mean of variances**	193.5		30.0		2.9	

Parameter estimates and local approximations of their uncertainty after the traditional, the first optimized, and the second optimized experiment. Shown are the system parameters which are believed to be identical from cell to cell: the degradation rate 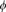 of 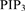, the dissociation rate 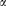 for the dissociation of Akt from the plasma membrane, the inhibitor constant 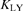 for LY29, and the association rates 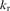 for the binding of iRap to the FKBP domain of CF-p85, 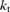 for the formation of the trimeric complex of CF-p85, iRap, and Lyn-FRB, and 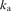 for the recruitment of Akt to the plasma membrane. 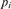 and 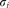 denote the parameter estimate and its corresponding uncertainty after the 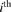 experiment was performed.

### Optimal experimental design for parameter estimation

In order to maximize the information gained about kinetic and pharmacological properties, we asked if we could find a second experimental protocol 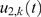 with given 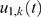 that would minimize the predicted parameter variances from a collective fit. Such a minimum would correspond to a smaller confidence region (shaded blue in Figure 1D). Figure 1G shows the ellipsoid approximation of this confidence region for a case with two parameters. Different geometric properties of this confidence ellipsoid correspond to properties of the covariance matrix 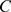. We chose to minimize the mean of the diagonal elements (the trace of 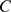 divided by the number of parameters) to balance the experimental effort between parameters. We would like to note that several other properties of 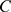 have been proposed as design criteria. For example, minimizing the determinant corresponds to minimizing the volume of the uncertainty region, whereas minimizing the maximum eigenvalue (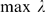) corresponds to minimizing the longest half-axis (Figure 1G).

**Figure 2:**
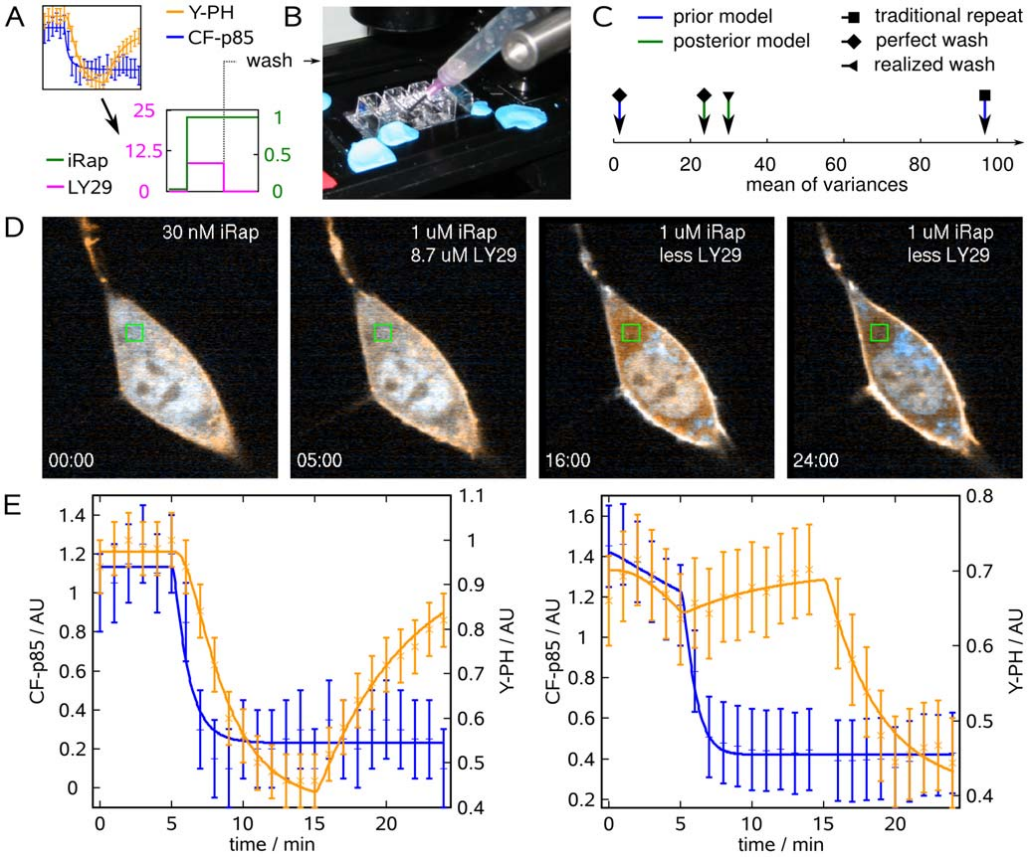
Numerical optimization of experimental design and practical experimentation to infer model parameters from live-cell microscopy data. (A) Schematic of sequential experimentation. The traditional design (left) resulted in a model that was used for optimizing the concentration-time profile of iRap (green) and LY29 (pink) for the next experiment. (B) The washout of LY29 at minute 15 was realized by inserting a needle connected to a vacuum trap, and rinsing with 5 ml of 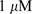 iRap in extracellular buffer. (C) Mean of parameter variances predicted for or achieved with the optimized experiment. Arrows indicate the parameter variance at different stages of model development, and under consideration of experimental uncertainty introduced by the wash. (D) Confocal fluorescence images of an NIH 3T3 cell (CF-p85 shown in cyan, Y-PH in yellow). 30 nM iRap was added immediately before triggering time-lapse acquisition. After 5 min, 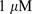 iRap and 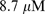 LY29 were added. 10 min later, LY29 was washed out, using 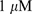 iRap in extracellular buffer. Experiment time is shown in minutes. (E) Combined parameter estimation from both the traditional (left) and optimized (right) experiment. Error bars show data, continuous lines show model output. Blue error bars and lines show cytosolic fluorescence of CF-p85, orange error bars and lines cytosolic fluorescence of Y-PH. The frame captured at minute 15 of the optimized experiment was lost to brightfield illumination during the washout procedure.

Using the trace criterion, the second experimental design 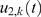 was optimized with the previous experiment 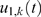 included in the prediction of 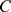 and based on the current parameter estimates. 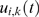 were piecewise constant functions that were discretized into three intervals. This means that the composition of extracellular buffer was allowed to change twice at 5 and 15 min with measurements taken every minute for 24 min. The maximum concentrations of drug allowed were 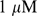 for iRap or 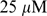 for LY29. The traditional experimental protocol served as an initial design that was optimized by a numerical method that employs in its core a sequential quadratic programming (SQP) method [20] (see Methods). SQP is an efficient derivative-based local optimization method that approaches the Karush-Kuhn-Tucker conditions (local optimality conditions) iteratively. It is particularly suited for large optimization problems with many design variables such as drug concentrations. How good a local minimum is approximated by an SQP run depends on termination critera. Typically, good improvements of the initial design can be observed in practice (see for example [8]). Here, we obtained from a single SQP run an experimental protocol (Figure 2A) for which the mean of variances was predicted to be more than 50-fold reduced when compared to a repetition of the established protocol. The numerically optimized protocol suggested that we add a low dose of iRap for the first 5 min. Then, the concentration of iRap should be increased to 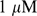 in the presence of 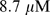 LY29 for the next 10 min. Finally, LY29 should be washed out in the presence of iRap (Figure 2B). While in general, the predicted improvement of parameter estimates is subject to parameter and model uncertainty, the predicted gain of information appeared promising (Figure 2C, blue arrows).

Figure 2D shows confocal microscopy images from the realization of this numerically optimized experiment. Translocation of CF-p85 was induced submaximally using 30 nM iRap for 5 min, but was then reinforced by the addition of 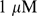 iRap in the presence of 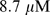 LY29. The addition of 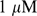 iRap induced rapid translocation of CF-p85 while Y-PH remained cytosolic due to the inhibitory effect of LY29 on PI3K. Ten minutes later, LY29 was washed out with extracellular buffer containing 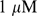 iRap. The buffer exchange of most of the solution occurred in less than one minute by making use of combined addition of buffer and vacuum suction (Figure 2B). Immediately after the washout of LY29, Y-PH started to translocate to the plasma membrane, indicating that the production of 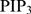 resumed (see also Video S2).

### Uncertainty in biological experimentation

The experiment illustrates an important problem that arises often from numerical optimization of biological experiments, in that these experiments are often challenging and introduce additional uncertainty. For example, the withdrawal of a growth factor stimulus may only partially reduce signaling due to tight receptor binding. Similarly, the knock-down of a protein by siRNA is never complete, and the magnitude of reduction in protein expression may vary from cell to cell. In this particular case, it was conceivable that some LY29 remained in the imaging chamber after the washout. While such experimental uncertainties are often irrelevant to traditional biology, the implications on parameter estimation from biological data can be significant. Thus, the implications of experimental imperfection on the performance of numerically optimized and idealized experiments need to be addressed.

In this specific case, we accounted for experimental uncertainty by estimating the residual concentration 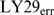 from the time course data as part of the parameter estimation problem. To this end, the concentration of LY29 (eq. 3) was redefined posteriorly:
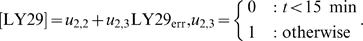
(8)The parameter estimates obtained from a combined fit to the traditional and the optimized experiment (Table 1) calibrated the model in excellent agreement with data (Figure 2E). While the estimate for 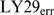 clearly suggested that residual LY29 was present, it did not significantly affect the large improvement of the resulting parameter estimates (Figure 2C, green arrows; Table S1).

### Constraining the complexity of experimental designs

A practical limitation in live-cell imaging experiments is that only a limited number of buffer changes can be performed without exerting cell stress. Instead of employing a fixed time-point model for adding drugs, it could also be beneficial to optimize at which time points drugs are added. We therefore derived a constraint formulation that extends the previous approach by optimizing the time points of two buffer changes in addition to their drug composition.

To this end, the system of ODEs was subjected to a time transformation, by which the differentials were multiplied by a piecewise constant control function 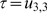 that was discretized into the three segments of incubation. In this parameterization, large values of 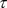 correspond to longer intervals between buffer changes with more measurements, which was reflected by transforming the error model accordingly to 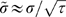. In addition, a dynamic constraint formulation was employed to ensure that the duration of the optimized experiment would equal to the duration of the established protocol:
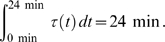
(9)Further, to facilitate the experiment, we limited the protocol to drug additions (see Methods). This also reflects a protocol constraint in high-throughput microscopy where wash-out procedures are difficult to implement.

**Figure 3:**
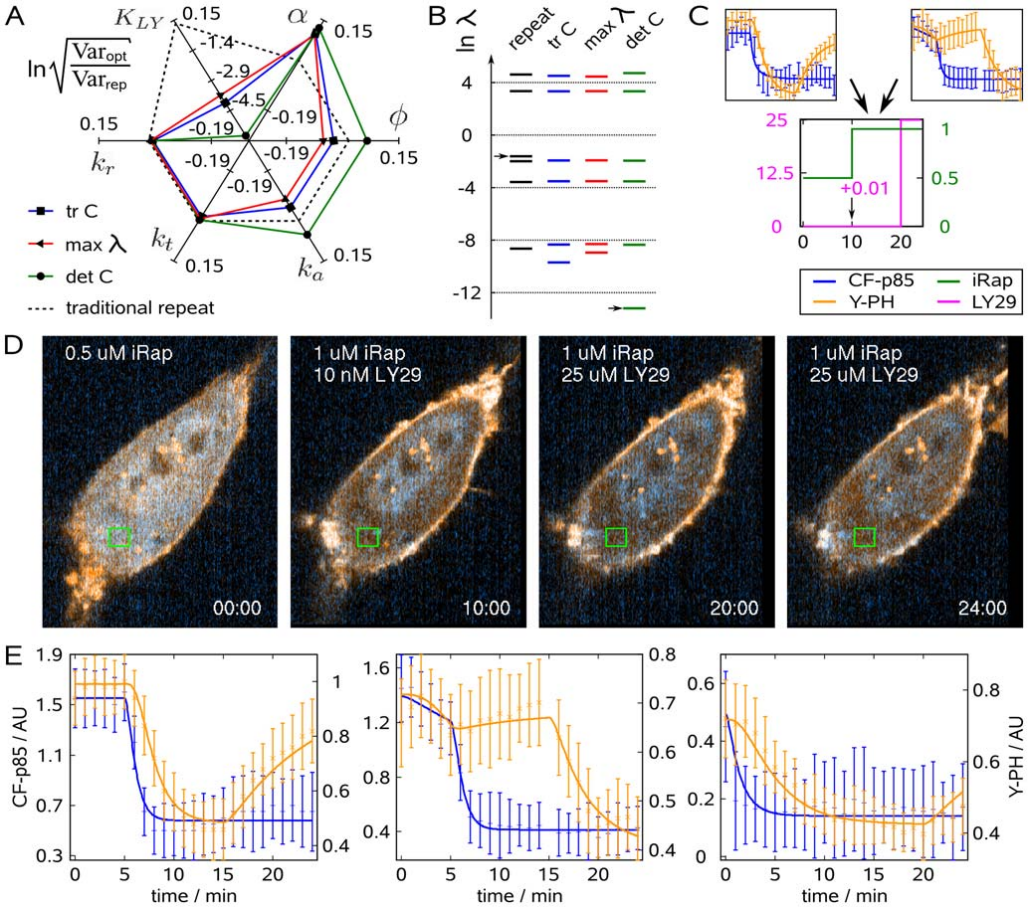
Minimization of different uncertainty criteria under experimentally meaningful design constraints. Different uncertainty criteria were minimized by finding optimal time points for adding drugs and optimal concentrations. Washouts were excluded from the design space by a dynamic constraint formulation. (A) Performance comparison of different criteria with respect to parameter uncertainty or (B) the eigenvalue spectrum of the optimized experimental design. Arrows indicate the eigenvalue that is aligned with the uncertainty of 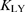. (C) Schematic of sequential experimentation. The data from the traditional and the first optimized experiment were used for optimizing the concentration-time profile of iRap (green) and LY29 (pink) for the next experiment. (D) NIH 3T3 cell (CF-p85 shown in cyan, Y-PH in yellow). 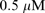 iRap was added at the beginning of the experiment. 10 min later, another 10 nM LY29 and another 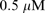 iRap were added. PI3K remained active as judged from low cytosolic fluorescence of Y-PH. 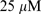 LY29 was added at minute 20, resulting in dissociation of Y-PH from the plasma membrane. Time is shown in minutes. (E) Combined parameter estimation on all three experiments. Error bars show data, continuous lines show model output. Blue error bars and lines show cytosolic fluorescence of CF-p85, orange ones show cytosolic fluorescence of Y-PH.

Under these constraints, experimental designs were optimized for different design criteria (Figure 3A, 3B) based on the diagonal elements, the maximum eigenvalue, and the determinant of 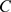. While for any experiment only small overall improvements of the parameter estimates were predicted when compared to a repetition of the established protocol, the three optimized designs mostly aimed at the uncertainty of 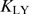. Such an opportunity for narrowing down selectively a single parameter would have been difficult to predict by intuition.

### Sequential model refinement from live-cell data

In this specific situation, we used the determinant criterion to optimize particularly for the inhibitor constant of LY29. According to this second optimized protocol (Figure 3C), cells were treated with 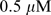 iRap for 10 min, eliciting fast translocation of CF-p85. In the course of 10 min, membrane-targeted CF-p85 stimulated the production of 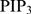, as was indicated by a decline of cytosolic fluorescence from Y-PH. The concentration of iRap was then doubled with a concomitant addition of 10 nM LY29. PI3K activity was largely insensitive to such a low dose of inhibitor, thus establishing a lower bound for 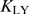. Ten minutes later, 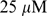 LY29 was added, triggering rapid dissociation of Y-PH (Figure 3D, Video S3).

Finally, we formulated the parameter estimation problem on the data obtained from the traditional and both optimized experiments. To obtain a satisfying collective fit for all three experiments, we considered background fluorescence from CF-p85 and Y-PH recruited to the plasma membrane and to internal membranes. For simplicity, a fraction 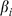 of bound CF-p85 and Y-PH was included into the observation functions:
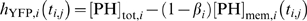
(10)

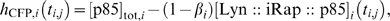
(11)Here the index 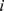 denotes the experiment number, 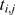 the time point 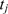 in experiment 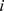. With this assumption, parameter estimation established good agreement between the model and the measurements from all three experiments combined (Figure 3E). Note that for 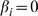, the revised observation functions revert to equations (4) and (5).

### Posterior assessment

**Figure 4:**
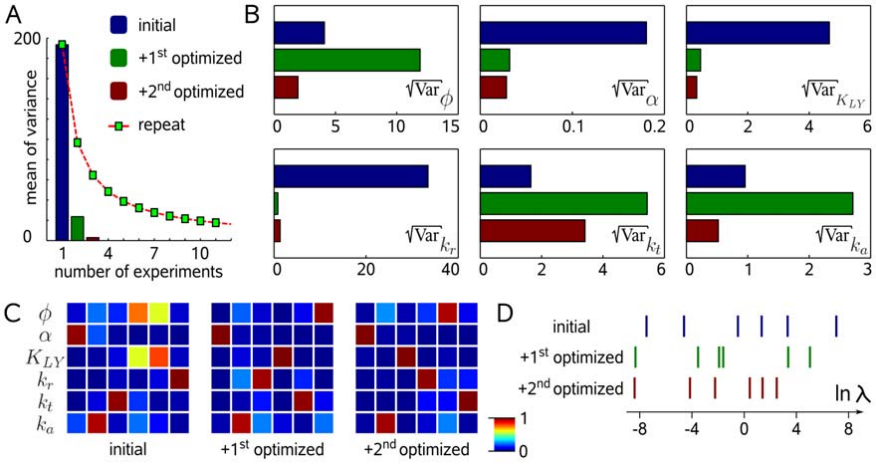
Decline of uncertainty. (A) The mean of parameter variances dropped from 193.5 after the traditional experiment to 2.9 after the second optimized one. For comparison, parameter uncertainties were calculated for repetitions of the intuitive traditional experiment. (B) Parameter uncertainties after individual experiments were performed. (C) Normalized eigenvectors of the corresponding covariance matrices ordered in ascending length suggest that correlation between biological parameters was largely eliminated. (D) The ranges of the eigenvalue spectra of 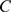 were consistently reduced.

To assess if optimal experimental design yielded data that reasonably defined the parameters of the proposed 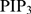 signaling model, we approximated the uncertainties of parameter estimates after each of the three experiments (Table 1). Overall, the mean of variances dropped from 193 to 2.9 (Figure 4A), much faster than one could expect from triplicate measurements. Most of this decline reflects improvements of the parameter estimates with the highest initial uncertainty, 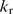, 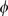, and 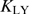. Moreover, the parameters controlling downstream signaling, 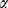 and 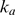 could be inferred with much improved accuracy. From experiment to experiment, however, the parameter estimates varied strongly, in agreement with most relative uncertainties remaining above 100%. Nevertheless, we identified a protocol that renders all endogenous parameters accessible in few single-cell experiments. This protocol of three designs could be refined further by taking advantage of the improved previous knowledge.

On the other hand, the uncertainty of the exogenous parameter 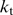 increased from the initial experiment to the final model because it describes the association of CF-p85 to Lyn-FRB. Lyn-FRB is not fluorescently tagged and is virtually unidentifiable with free 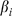 (Table S1). Consistently, 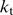 is the least defined system parameter in the final model (Figure 4B). Nevertheless, this uncertainty does not propagate to endogenous parameters because the localization of CF-p85 is observable. This is a favorable property because it suggests that the exact transfection efficiency for Lyn-FRB is not critical as long as it expresses much better than CF-p85.

In their study, Gutenkunst *et al.* used published intuitively designed experimental protocols to characterize parameter identifiability in different models [6]. Independently of the size of these models, they found that a substantial fraction of eigenvectors of the uncertainty region ran along multiple axes in parameter space. The eigenvectors of 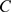 computed after the intuitive experiment from Figure 1 suggested a similar co-dependence also for this model (Figure 4C). However, the data from a single numerically optimized experiment largely eliminated the correlation between estimates. The corresponding eigenvalue spectrum of 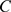 confirms that also the extent of the uncertainty region was consistently reduced (Figure 4D).

## Discussion

An important goal of signal transduction research is to obtain molecularly explicit mathematical models that describe the flow of information. A particular challenge in obtaining such models is the large number of poorly defined parameters. Here, we demonstrate for the first time the use of an optimal control method to interrogate single cells for those parameter values by sequential optimization of experimental designs. Our results suggest that this approach relies on four main ingredients: (i) a carefully parameterized dynamic model, (ii) quantitative measurements with appropriate error assumptions, (iii) pharmacological perturbation tools, (iv) and an experimental design space that is realistically constrained. We show that an efficient numerical algorithm can derive non-intuitive experimental designs that significantly reduce the experimental effort to infer parameters of a dynamic model with good statistical quality.

### Using optimal experimental design for parameter estimation in cell signaling

Our study focused on how an optimal control method can be employed to interrogate a cell signaling system by live-cell microscopy. The goal of this approach was to reveal biologically relevant parameters of a model that describes the dynamic behavior of the 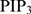 lipid second messenger, the recruitment of a cytosolic pleckstrin homology domain to the plasma membrane, and the inhibition and activation of PI3K by means of pharmacological and synthetic biology techniques. We found that the data obtained from the established experimental protocol resulted in large parameter variances and, in effect, inconclusive estimates. However, the model was useful to inform an optimal control method that proposed modifications of the existing protocol to markedly reduce parameter uncertainty. While we found that the appropriate parameterization of the design space is important for the attainability of optimized concentration-time profiles, experimental imperfection led only to a small reduction in information gain (Figure 2C). On the other hand, the uncertainty of parameter estimates appeared to be more important (Figure 2C), suggesting that methods for robust optimal experimental design will be of much practical value [15]. Importantly, not only robustness to uncertainty of parameter estimates, but also to uncertainty in model structure and experimentation will need to be addressed more formally by future optimization methods, e.g. along the developments described in [21].

Considering the specific model structure of the translocation assay, the first optimized experiment at a first look did not seem intuitive. However, it can be rationalized as an intelligent maneuver to separate the translocation kinetics of CF-p85 from the production of 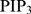. To this end, a high concentration of iRap was added in the presence of the inhibitor LY29, and then LY29 was washed out after CF-p85 was brought into position. By employing a submaximal dose of LY29, the identifiability of the inhibitor constant could be improved simultaneously. This suggests that the first optimized experiment clearly exploited features of the experimental system that were independent of details in model structure and parameter values. Moreover, it is interesting to note that for the last segment of the experiment, it was not necessary to wash out LY29 completely. Our posterior analysis that accounted for experimental imperfection suggests that significant residual amounts of LY29 did not significantly affect the realized design performance (Figure 2C).

The second optimized experiment employed novel dynamic constraint formulations to optimize a limited number of buffer changes and time points. These formulations could be of general value for reflecting actual experimental constraints. With the constrained design spaces used for optimizing the second experiment, we were limited to three additions of drug. While this design was clearly informative, its performance with respect to parameter identifiability was sensitive to uncertainty of 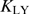. This exemplifies a drawback of minimizing the determinant of the covariance matrix. Since the determinant criterion aims at the volume of the confidence ellipsoid (Figure 1G), an experimental design that defines a single parameter value such as 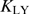 very tightly, would correspond to large improvements in the objective function. In other words, minimizing 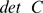 in some cases focuses experimental effort at eliminating a full dimension of the uncertainty region (Figure 3B). Thus, despite the significant improvement predicted for 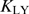, it should be noted that focusing effort on a single parameter can be sensitive to overall uncertainty.

### Enhancing the identifiability of endogenous parameters by chemical biology

The practical non-identifiability of many parameters is an important challenge for the development and use of signaling models. Intuitively, this means that there are too many parameters for the number and the type of experiments that can be practically performed. Moreover, the structure of such models was suggested to reflect natural robustness to changes in many parameter values [3],[6]. For example, signaling pathways often translate graded stimuli into all-or-none responses [22], such that downstream observations become insensitive to upstream kinetic parameters. Strong covariance between parameters of reaction cascades, fast reversible reactions, and binding events makes it difficult to constrain single parameter values [3],[6],[10],[23]. Therefore, pharmacological and synthetic tools that drive a biological system internally will become important for eliciting informative dynamics. The pair of Lyn-FRB and CF-p85 is one example of such tools, because endogenous PI3K is activated directly and downstream of complex receptor relays. Likewise, the small molecule inhibitor of PI3K that we employed exemplifies the value of pharmacological tools in the context of such perturbation studies. In essence, our work suggests that chemical tools may be useful for dividing complex pathways into tractable signaling domains in order to conquer non-identifiabilities *in situ*. However, it was not clear intuitively how to combine these perturbations in order to generate data from which parameters could be inferred. Our work exemplifies that in such a situation, model-based experimental design can dramatically reduce the experimental effort to establish a useful protocol.

### Challenges to parameter estimation in systems biology

To summarize, several issues should be considered when a signaling system is explored using a dynamic model. First, it is often difficult to develop comprehensive cell signaling models that are simple enough to be useful but that do retain enough detail to represent the essential features of a system quantitatively. Finding a trade-off between overparameterized model formulations and formulations that neglect important interactions is made difficult by our limited knowledge of the mammalian signaling network and by often large uncertainties in measurements. Second, our study suggests that pharmacological perturbations are powerful tools to enhance the quality of parameter estimates. We show that when such pharmacological agents are not available, synthetic biology approaches can be employed. In all those cases, numerical methods for optimal experimental design can propose protocols that take advantage of these experimental techniques. Third, the experimental design space should be constrained during optimization to result in feasible protocols. This ensures that the experiment can be realized according to the proposed plan. At the same time, our results suggest that some experimental protocols can be robust to imperfection (Figure 2C). Our approach to reveal parameter values of biological models complements other approaches of optimal experimental design in systems biology, such as designing experiments for model discrimination [24] or for minimizing prediction uncertainty [25].

### Pharmacological, biochemical, and clinical cell signaling assays

Ideally, the parameter values of a biochemical model correspond to biologically meaningful properties. In our example, 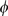 reflects the degradation rate of 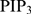, 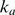 and 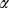 describe the affinity of a PH domain, and 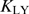 reflects the inhibitor constant of LY29. Because any model can only be an approximation of the true process in nature, the parameter values we determine are model-specific. Nevertheless, if such parameters could be inferred with sufficient accuracy, they may reflect molecular differences that underlie biochemical mechanisms. We could envision that in a pharmacological context, drug candidates for targeting PI3K could be compared *in situ* by inferring 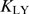 from few single-cell experiments. Likewise, in a biochemical context, differences in PH domain affinities could be revealed by estimating 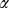. Finally, 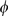 reflects the activity of phosphatases like phosphatase and tensin homolog (PTEN) which is lost in many cancers [26]. Importantly, inferring 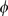 in cell lines established from cancer patients could reveal changes in PTEN activity that originate from causes beyond gene expression. To achieve this, instead of minimizing a criterion on the full covariance matrix (e.g. the trace or determinant), experimental effort could focus only on the subset of diagonal elements of interest. Applications of such an approach may include a comprehensive estimation of signaling parameters from cancer samples, possibly leading to individualized therapy. Also, by treating drug action as a systems property, this approach may also be useful to evaluate possible off-target effects of drugs.

### Conclusion

We showed here for the first time that optimal experimental design for parameter estimation yields rich data for rapid model development in systems biology. This approach aims to restrict the range of possible parameter values, and is therefore a suitable way to challenge the validity of a model by quantitative experimentation. In particular, the results exemplify the use of this optimal control method to interrogate individual cells for pharmacological and biological parameters that underlie a growth-related second messenger signaling system.

## Materials and Methods

**Cell culture, constructs, and drugs.** 4-well LabTek chambered coverslips (nunc, Rochester, NY) were coated with 0.1 mg/ml poly-L-lysine (Sigma-Aldrich, St. Louis, MO) in hydroborate buffer. 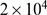 NIH 3T3 cells were seeded per well, and grown close to confluency. Per well (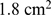), 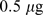 of each construct (Lyn-FRB, CF-p85, Y-PH) were transfected using 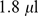 Lipofectapine 2000 in 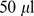 total transfection mix on 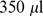 DMEM supplemented with 10% FBS for 6 hrs (all reagents from Invitrogen, Carlsbad, CA). 4 to 12 hours prior to imaging, cells were serum-starved in 0.1% BSA (Sigma-Aldrich) in DMEM. CF-p85 is described in [17], and Lyn-FRB (as 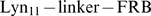) in [27]. Stock solutions of LY294002 (Tocris Bioscience, Ellisville, MO) and iRap were prepared in DMSO. For the organic synthesis of iRap from rapamycin and 3-methylindole, see [27].

**Numerical methods.** The numerical methods used are incorporated in the software package VPLAN [7],[8]. They include a variable–step variable–order BDF method for initial value problems in differential equations, which is used for the simulation of the translocation model. This solver is based on [28],[29] and computes first and second derivatives of the solution of the initial value problem with respect to initial values and parameters, utilizing a sophisticated combination of internal numerical differentiation [30] and automatic differentiation [31]. Parameters in the translocation model were estimated using a multiple shooting method with a generalized Gauss-Newton algorithm that is implemented in PARFIT (see [30],[32], reviewed in [33]). Multiple shooting methods consider the parameter estimation problem as a least squares problem that is constrained by a differential equation model. The differential model constraint is discretized like a boundary value problem. This enhances stability of the method and allows for initialization of the discretized output trajectories close to measurement data, which results in fast convergence to statistically stable minima. In contrast, single shooting methods only solve the initial value problem from the beginning of an experiment, and integrate with possibly poor initial guesses for parameter values along the full time axis. If at all feasible, a solution is found only with much more effort than by multiple shooting. Recently, Balsa-Canto *et al.* proposed a hybrid approach that switches from a global search to a multiple shooting Newton-type method [34]. Optimal experimental designs are determined by minimizing a function of the covariance matrix of the parameter estimation problem (e.g. the sum of the diagonal elements) with respect to the concentration-time profile of drugs and possibly the sampling scheme. The method is applicable to constrained parameter estimation problems in systems of differential algebraic equations (DAE). In the general case of constrained problems, the covariance matrix 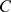 can be represented in terms of 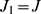 (see eq. 7) and the sensitivities 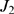 of the constraints:
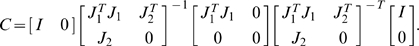
(12)To exclude washout procedures in the second optimized experiment, we defined a differential state variable 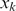 as a “short-term memory” for each drug, and defined diffusion-like equilibration with the current concentration 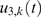:
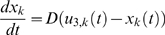
(13)By introducing the dynamic inequality constraint 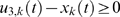 which needs to be evaluated only once after each buffer change, we can approximate a constraint of the form 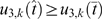 for 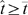. The constraint is stricter the larger one choses 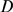. In our specific case, 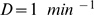 was sufficient. These constrained optimal control problems were solved by a direct approach for optimal control, the core of which is solving a structured nonlinear program by SQP-type methods [20]. All calculations were performed on a Dell Optiplex GX620 workstation.

**Live cell microscopy.** Cells were imaged at 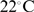 using a UApo/340 40x/1.35 oil objective (Olympus, Center Valley, PA) mounted on an Olympus IX70 stage customly equipped with an UltraVIEW spinning disk confocal scanner (PerkinElmer, Waltham, MA). CF-p85 and Y-PH were excited with a He-Cd laser (IK series from Kimmon, Centennial, CO) or an Ar laser (model 60B from ALC, Salt Lake City, UT), using 442/10 or 515/10 excitation filters, and 480/40 or 530LP emmission filters, respectively. The variable power supply of the Ar laser was taped at the beginning of this study, and the power of each laser was measured on stage through an Olympus UPlanFl 10x/0.30 objective before every experiment using an optical power meter (model 835 from Newport, Irvine, CA) to safeguard quantitative analysis. Images were captured from a Hamamatsu C4742 Orca (Hamamatsu Photonics, Shizuoka, JP) at 12 bit/pixel and 300 msec exposure time. Shutters were emulated using a closed position of the excitation filter wheel controlled by a Sutter Lambda 10–2 (Sutter Instrument Company, Novato, CA). Extracellular buffer contained 5 mM KCl, 125 mM NaCl, 1.5 mM 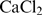, 1.5 mM 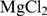, 10 mM D-glucose (Sigma-Aldrich), and 20 mM HEPES (Invotrogen).

**Image analysis.** For each experiment, an empty region of the coverslip was captured in both fluorescence channels, and used for background subtraction. Image stacks were aligned with subpixel resolution to compensate for planar drift where needed. Fluorescence was quantified using the median of a region of 25
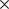
25 pixels, that was chosen to minimize interference from passing organelles, and that is marked by green squares in Figures 1B, 2D, and 3D. The standard deviation of measurements 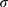 was obtained from the standard deviation of intensities in the region of 25
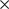
25 pixels for each frame. For optimizing designs, we assumed a constant error model, however with larger errors for CF-p85 (0.2 AU) than for Y-PH (0.06 AU).

## Supporting Information

Table S1 Estimates for calibration parameters.
(0.11 MB XLS)

Text S1 Model represented in SBML layer 2 version 2, featuring two compartments (the cytosol and the plasma mebrane) and all molecular species, including iRap and LY29. The parameter values are set to the estimates from the collective fit to all data.
(0.01 MB XML)

Video S1 Initial Experiment
(0.89 MB MOV)

Video S2 First optimized experiment
(1.00 MB MOV)

Video S3 Second optimized experiment
(1.32 MB MOV)
